# A wear-resistant coating with combined mechanical and antifouling properties for potential underwater cleaning applications

**DOI:** 10.3389/fchem.2025.1666881

**Published:** 2025-09-16

**Authors:** Xiping Chen, Leika Du, Jiawang Chen, Huanzhi Xu, Qinghua Zhang, Jiankun Hu

**Affiliations:** 1 Marine Science and Technology College, Zhejiang Ocean University, Zhoushan, Zhejiang, China; 2 Zhejiang Feijing New Material Science & Technology Co., Ltd., Zhoushan, Zhejiang, China; 3 Donghai Laboratory, Zhoushan, Zhejiang, China; 4 College of Chemical and Biological Engineering, Zhejiang University, Hangzhou, Zhejiang, China

**Keywords:** silicone, low-surface-energy, antifouling, underwater cleaning, A/MoS2/PTFE

## Abstract

**Introduction:**

Silicone-based low-surface-energy antifouling coatings are environmentally friendly, but their widespread application is hindered by the inherent challenge of achieving a balance between mechanical durability and antifouling efficacy.

**Methods:**

This study developed a novel multifunctional anchoring material, N,N’-bis(12-hydroxystearoyl)-1,3-phenylenediamine (A), via a condensation reaction. Silicone antifouling coatings were then synergistically modified with A, molybdenum disulfide (MoS2), and polytetrafluoroethylene (PTFE), followed by room-temperature crosslinking to form a composite coating.

**Results:**

The incorporation of 1% A significantly enhanced the coating’s performance: surface roughness was reduced by 33% (from 1.12 μm to 0.75 μm), the water contact angle increased from 118.2° to 122.7°, and tensile strength was improved by 85% (from 1.08 MPa to 2.00 MPa). The elastic modulus increased by 130%, while underwater friction decreased by 64% (from 2.41 ± 0.09 N to 0.87 ± 0.04 N). The coating demonstrated exceptional durability, with an average surface roughness (Sa) remaining below 2.65 μm after 2000 abrasion cycles. Furthermore, it exhibited outstanding self-cleaning efficiency (>97.1 ± 0.87%) and antibacterial rates (>94.5 ± 1.78%). Marine field tests confirmed effective antifouling performance for over 90 days during peak fouling season.

**Discussion:**

The synergistic effect of A, MoS_2_, and PTFE successfully overcame the key limitations of traditional low-surface-energy coatings—poor mechanical strength and weak wear resistance. This work provides a breakthrough in designing high-performance, durable antifouling coatings with strong potential for practical applications, particularly in underwater cleaning robotics.

## Graphical Abstract

**Figure:**
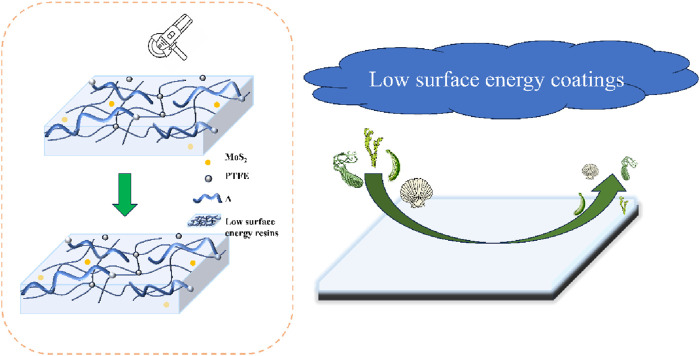

## 1 Introduction

The shipping industry serves as a critical enabler of global trade and economic integration (Zhou et al., 2023). However, marine biofouling poses significant challenges, including hull structural degradation, elevated fuel consumption, and the introduction of invasive species into non-native ecosystems (Iswadi et al., 2022; Thach and Van Hung, 2024). To address these issues, various antifouling strategies have been explored, such as electrochemical treatments (Deng et al., 2025), ultrasonic cleaning (Zhong et al., 2022), mechanical removal (Chen et al., 2023), and antifouling coatings (Deng et al., 2021; Xu et al., 2025; Yang et al., 2025). Among these, antifouling coatings are widely regarded as one of the most economically viable solutions (Zang et al., 2024).

Zwitterionic polymer antifouling coatings and biomimetic antifouling coatings have emerged as prominent research focuses (Dai et al., 2024; Liu et al., 2024; Murali et al., 2024; Roeven et al., 2021)). However, their commercialization remains challenging due to high production costs and complex fabrication processes (Chen et al., 2021; Muadtrap et al., 2025). In contrast, silicone-based low-surface-energy coatings, characterized by mature preparation techniques, long-term antifouling efficacy, and eco-friendly advantages, are considered a key development direction in marine antifouling technologies (He et al., 2023).

Furthermore, regulatory requirements for pre-port hull cleaning in countries such as Australia and New Zealand have imposed additional demands on antifouling coatings (Xavier et al., 2025). To meet underwater cleaning standards, it is essential to develop coatings that combine low surface energy with wear resistance. However, existing low-surface-energy coatings often suffer from insufficient mechanical strength, leading to coating damage during cleaning processes and compromised antifouling performance, thereby limiting their application in intelligent cleaning systems (Chu et al., 2024).

To overcome this technical hurdle, numerous research efforts have been undertaken. For instance, Liu et al. incorporated lignin into superhydrophobic coatings, demonstrating remarkable wear resistance with water contact angles maintained above 150° after 30 sand abrasion cycles (Liu et al., 2022). Zhang et al. achieved significant improv-ments in coating durability by introducing PDMS, enabling the material to maintain excellent integrity even after 200 friction cycles (Zhang et al., 2025). Similarly, Xiang et al. enhanced the flexibility of textile-based coatings by incorporating nano-ZnO/PVSQ composites, resulting in an 18% increase in fracture elongation (Feng et al., 2024). Further advancing this field, Sun et al. developed hydrophobic Al_2_O_3_/SiO_2_/PDMS composite coatings, which exhibited a remarkable 250% improv-ment in fracture elongation (Sun et al., 2022). Wang et al. developed carbon nanotube-reinforced PTFE coatings exhibiting exceptional hydrophobicity (154.1° ± 2° contact angle) that retained their superhydrophobic properties even after 500 abrasion cycles under 50 g/cm^2^ pressure (Wang et al., 2017). In another approach, Liao et al. significantly enhanced the tribological performance of polyetheretherketone (PEEK) by introducing graphite/PTFE composites, achieving an 80% reduction in friction coefficient and an order of magnitude decrease in wear rate under 10 N loading conditions (Liao et al., 2022). However, these modifications failed to simultaneously optimize wear resistance, flexibility, and fouling resistance while introducing particle agglomeration issues. Guru et al. demonstrated that incorporating 3% molybdenum disulfide (MoS_2_) could effectively improve both tribological and antifouling properties of PTFE coatings, though the dispersion challenges of MoS_2_ remained unresolved (Guru et al., 2024). To improve filler dispersion, Yamada et al. employed 12-hydroxystearic acid (12-HSA) as a dispersant for carbon powder, while Meng et al. achieved superior dispersion of cobalt nanoparticles in octane using 12-HSA compared to stearic acid (SA) and oleic acid (OA) (Lu et al., 2005; Meng et al., 2013; Yamada et al., 2015). Although 12-HSA can mitigate particle agglomeration through hydroxyl-alkyl chain synergy, its gelation effect adversely affects coating viscosity and storage stability (Fameau and Rogers, 2020). To date, a universally effective solution to these challenges remains elusive.

This study synthesized a multifunctional anchoring material, N,N′-Bis(12-hydroxystearoyl)-1,3-phenylenediamine (A), via condensation reactions. The structure of A was characterized by FT-IR and ^1^H NMR. Subsequently, A was blended with MoS_2_ and PTFE, then dispersed into the silicone resin system. Room-temperature crosslinking produced a novel, mechanically robust antifouling coating. The results showed that adding 1% A reduced surface roughness by 33% (from 1.12 μm to 0.75 μm), increased the contact angle from 118.2° to 122.7°, enhanced tensile strength by 85% (from 1.08 MPa to 2.00 MPa), and improved elastic modulus by 130%. Additionally, underwater friction decreased by 64% (from 2.41 N to 0.87 N), and after 2,000 abrasion cycles, the average surface roughness (S_a_) remained below 2.65 μm. All coatings exhibited >97.1 ± 0.87% self-cleaning efficiency and >94.5 ± 1.78% antibacterial rates. Field tests demonstrated over 90 days of antifouling efficacy during peak biofouling seasons. The synergistic interaction of A, MoS_2_, and PTFE addressed the key limitations of traditional low-surface-energy coatings—insufficient strength and poor wear resistance. This work provides a breakthrough for antifouling applications and new insights into coating design for underwater cleaning robotics.

## 2 Experimental section

### 2.1 Materials

12-Hydroxystearic acid (12-HSA, ≥98%, Shanghai Titan Scientific), m-phenylenediamine (PDA, ≥99%, Aladdin), molybdenum disulfide (MoS_2_, ≥99.5%, Aladdin), polytetrafluoroethylene powder (PTFE, ≥99%, Aladdin), and hydrogen-terminated silicone oil (821, viscosity 50 Pa·s, Quzhou Haina) were used as primary materials. Other chemicals included xylene (Xy, analytical grade), azobisisobutyronitrile (AIBN, analytical grade), dibutyltin dilaurate (DBTDL, catalyst grade), Polydimethylsiloxane (PDMS, 50 mPa·s) and copper (II) chloride dihydrate (CuCl_2_·2H_2_O, reagent grade), all sourced from Aladdin.

### 2.2 Preparation of N,N′-Bis(12-hydroxystearoyl)-1,3-phenylenediamine (A)

**SCHEME 1:**
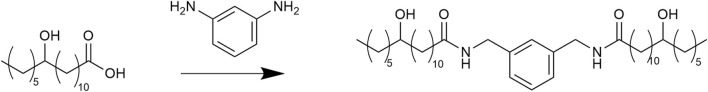
Synthesis of N,N′-Bis(12-hydroxystearoyl)-1,3-phenylenediamine (A).

Under an anhydrous argon atmosphere, 12-hydroxystearic acid (12-HSA, 150.27 g) was melted at 130 °C m-Phenylenediamine (PDA, 34.05 g) was then added dropwise to the molten 12-HSA over 6 h under continuous stirring. The reaction mixture was maintained at 130 °C until the amount of condensed water reached 95%–98% of the theoretical value (calculated for complete amidation), affording N,N′-Bis(12-hydroxystearoyl)-1,3-phenylenediamine (A) as a pale yellow waxy solid. It is used after recrystallization of ethanol twice. The synthetic pathway is depicted in Scheme 1. Table 1 is the formulation and composition of coatings with varying A, MoS_2_ (M), and PTFE (F) contents.

**TABLE 1:** Coating composition design matrix.

Coating	A_X_M_3_F_3_	A_1_M_3_F_Y_	A_1_M_Z_F_3_
A/g	8X	8	8
F (PTFE)/g	24	24	8Z
M (MoS_2_)/g	24	8Y	24
821/g	392-8X	408-8Y	408-8Y
PDMS/g	40	40	40
Xy (xylene)/g	320	320	320

Subsequently, A, MoS_2_, and PTFE were mixed with hydrogen-terminated silicone oil (821), PDMS, and xylene solvent according to the proportions specified in Table 1. The mixture was ground for 1 h under condensate water cooling and argon protection, filtered, and then the crosslinking agent DBTDL was added. The resulting mixture was cured at room temperature for 48 h to ultimately form the composite coating.

A: N,N′-Bis(12-hydroxystearoyl)-1,3-phenylenediamine, a synthesized anchoring material en-hancing mechanical and antifouling properties.

821: Hydrogen-terminated silicone oil (viscosity 50 Pa s), used as the base resin for crosslink-ing.

PDMS: Polydimethylsiloxane (50 mPa s), added at 10 wt% to regulate coating rheology.

Xy: Xylene, an organic solvent for uniform dispersion of components.

Subscripts (X, Y, Z) denote variable weight percentages of A, PTFE, and MoS_2_, respectively, as detailed in the experimental design.

### 2.3 Coating swelling ratio

The gravimetric method was used to monitor the *in situ* dissolution of each coating. Each slide was weighed before and after coating to determine the initial dry weight (W_dry_) of each sample. Three slides were used for each coating to obtain the mean and deviation. The coated slides were immersed in artifical seawater (ASW, prepared according to ASTM D 1141) containers that were changed every other day and weighed after a certain period of time (W_wet_). Both W_dry_ and W_wet_ needed to be adjusted by subtracting the initial weight of the slides (W_0_).

The swelling behavior of coatings was quantitatively evaluated using a gravimetric method. Prior to testing, glass slides were precisely weighed (W_0_) using an analytical balance (±0.1 mg). After coating application, the initial dry weight was determined by subtracting W_0_ from the coated slide weight. For statistical reliability, triplicate samples were prepared for each coating formulation.The swelling ratio (Q) was calculated as

$$Q=\frac{W_{\text{wet}}-W_{0}}{W_{\text{dry}}}$$

providing a quantitative measure of the coating’s water absorption characteristics.

The coated slides were immersed in ASW contained in sealed vessels. The ASW was renewed every 48 h to maintain consistent ionic strength. At predetermined intervals, samples were carefully removed, surface-dried with lint-free tissue, and immediately weighed to obtain the wet weight.

### 2.4 Coating surface morphology

The soaked slides were subjected to 2000 cycles of abrasion under a reciprocating friction and abrasion tester (220 N load, steel wool), and the coated surfaces before and after the abrasion were observed through high-depth microscopy VHX-6000.

### 2.5 Surface wettability

Static water contact angles were measured using a Kruss DSA100 goniometer (25 °C ± 0.5 °C, 50% ± 5% RH) following ASTM D 7334 standards. A precision microsyringe deposited 2 μL deionized water droplets (Milli-Q, 18.2 MΩ cm) vertically onto the cured coating surface. Droplet profiles were captured at 1,000 fps using an integrated high-speed camera, with the instrument software applying Young–Laplace fitting to determine contact angles. Five measurements were taken at different surface locations and averaged to ensure statistical significance (reported as mean ± standard deviation).

### 2.6 Underwater friction and wear testing

The coating solution was applied onto glass slide substrates and allowed to cure for 48 h. The cured samples were then immersed in artificial seawater for 24 h prior to testing. The coefficient of friction was measured using a tribometer equipped with an underwater testing chamber under a 310 N normal load.

The coating was uniformly applied onto standard glass slides and cured for 48 h. Wear resistance was evaluated using a reciprocating friction tester, where the coated samples underwent 2,000 abrasion cycles at a frequency of 60 cycles per minute (1 Hz) under a 220 N normal load, with Grade #0000 steel wool as the abrasive counterpart.

In this study, the mechanical stability of the coatings was evaluated by examining the correlation between the coefficient of friction and wear rate (Yan et al., 2016; Zhao and Zou, 2019).

$$W_{m}=\frac{m}{\rho \cdot F_{n}\cdot S}$$

Δm: mass difference before and after wear (mg, accuracy ±0.1 mg); ρ: coating density; Fn: load (N); S: sliding distance (cm).

### 2.7 Mechanical property tests

The coating solution was uniformly cast into polytetrafluoroethylene (PTFE) molds (50 × 10 × 1 mm^3^) and cured at room temperature for 48 h. After demolding, the resulting specimens were precisely cut into standard Type V dumbbell-shaped tensile bars compliant with ASTM D 638. Tensile properties were characterized using a universal testing machine (Instron 5,966 series) at a crosshead speed of 5 mm/min under ambient conditions (23 °C ± 2 °C, 50% ± 5% RH). The tensile strength and elastic modulus were calculated from the stress-strain curves, with five replicates tested for statistical reliability.

### 2.8 Self-cleaning tests

The self-cleaning property was assessed using copper (II) chloride dihydrate (CuCl_2_·2H_2_O) particles as model inorganic foulants. The particles were deposited on coated surfaces and subsequently removed with deionized water droplets to evaluate cleaning efficiency.

### 2.9 Antimicrobial performance test

Antimicrobial performance against *Escherichia coli* and *Staphylococcus aureus* was examined via plate counting. Coated samples were sterilized, immersed in bacterial suspension (10^8^ CFU/mL), and incubated at 37 °C for 5 h. Surface-adhered bacteria were collected by ultrasonic agitation, serially diluted, plated on LB agar, and quantified using ImageJ software.

### 2.10 Anti-fouling test for shallow sea mounted panels

The antifouling capacity test is carried out in accordance with the national marine antifouling coatings shallow sea experimental standard. Each coating was apply on the substrate, 300 × 250 × 3 mm^3^, at the bottom was epoxy zinc rich primer, and the finish coating was our experimental antifouling paint as prepared for testing. Three sets of parallel samples were prepared for each coating to test their antifouling capability at different depths in the ocean. After coating, the four corners of samples were fixed on the hanger with stainless steel bolts. After the installation completed, the hanger was placed on the experimental floating raft in the testing sea area. The experimental sea area is located in the offshore of Louman’s sheltered bay, Zhoushan, subtropical sea, the velocity of seawater is lower than 5 m/s, the azimuth is 30 ^o^ 01′N, 122 ^o^ 06′E, the test depth: 0.3–2.0 m, the testing lasted for 90 days, the main fouling organism attached to the board are algae and silt.

## 3 Results and discussion

### 3.1 Structural spectral analysis of A

**FIGURE 1:**
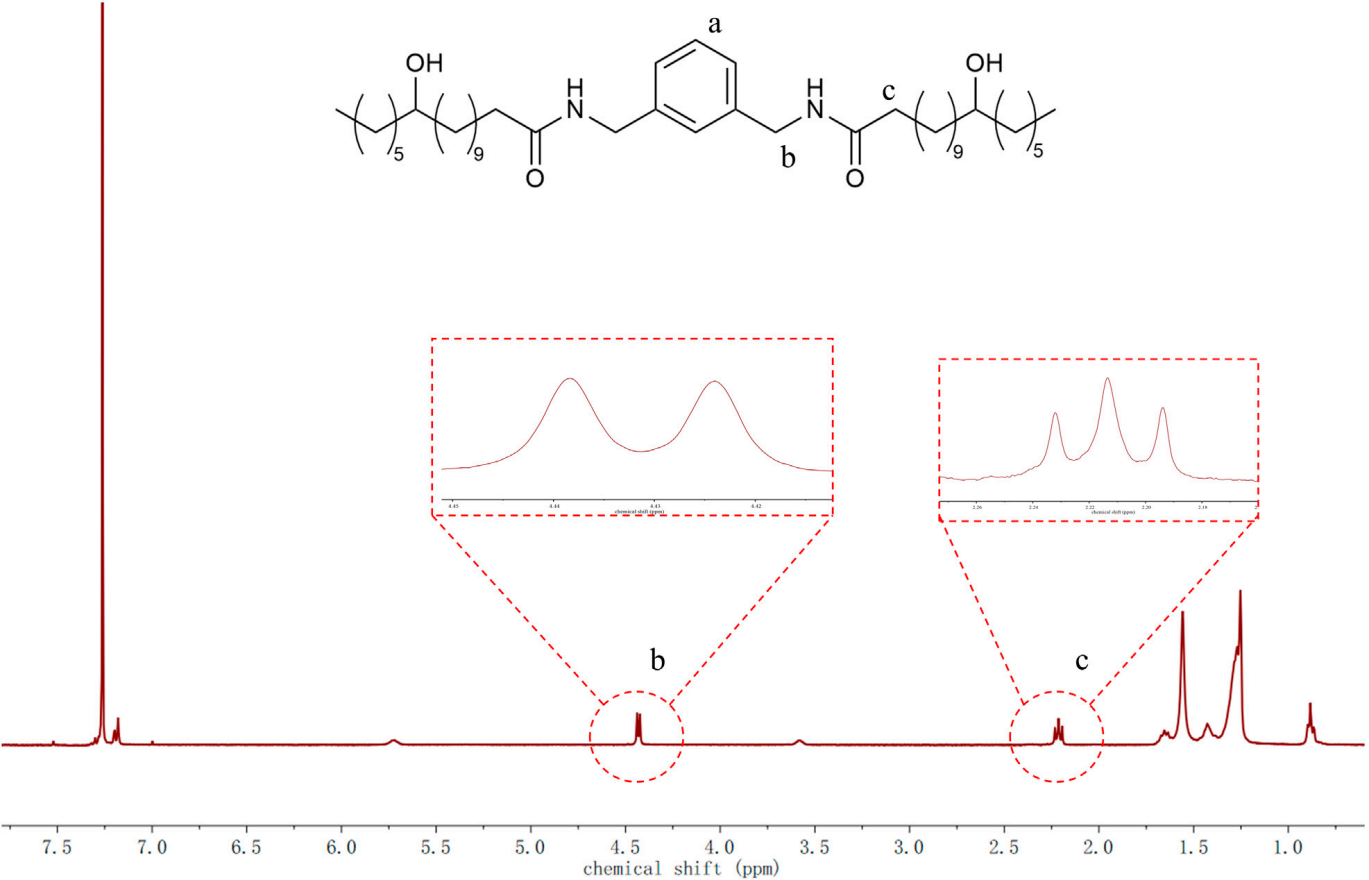
^1^H NMR spectrum of A with expansions. Proton designations in the structure **(a–c)** correspond to: aromatic **(a)**, benzylic **(b)**, and α-carbonyl methylene **(c)** environments. Expanded views are provided for regions b and c.

As shown in Figure 1, the ^1^H NMR spectrum of the synthesized compound A revealed characteristic proton signals at:7.20–7.40 ppm (multiplet, ^4^H, aromatic protons), 4.40 ppm (doublet, ^2^H, benzylic CH adjacent to amide group), 2.20 ppm (triplet, ^2^H, α-carbonyl CH_2_), 1.20–1.60 ppm (multiplet, ^31^H, methylene protons), 0.88 ppm (triplet, ^3^H, terminal methyl group). The hydroxyl proton signal was not detected, likely due to hydrogen bonding-induced peak broadening. All observed chemical shifts and splitting patterns were consistent with the expected structure of A.

**FIGURE 2:**
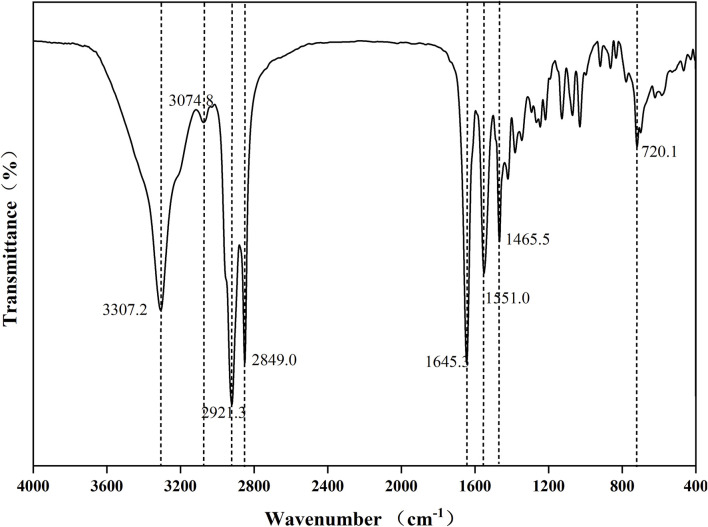
FTIR spectroscopic analysis of A.

As shown in Figure 2, FTIR spectroscopy confirmed the formation of amide bonds with characteristic absorption bands at:3,307 cm^-1^ (N-H stretching vibration), 2,849–2,921 cm^-1^ (C-H stretching of aliphatic chains), 1,645 cm^-1^ (C=O stretching of amide I band), 1,551 cm^-1^ (amide II band, coupling of N-H bending and C-N stretching), 720 cm^-1^ (aromatic C-H out-of-plane bending).The combined NMR and FTIR data conclusively demonstrated the successful amidation reaction between m-phenylenediamine and 12-hydroxystearic acid to form the target compound A.

## 3.2 Dissolution rate of coatings

**FIGURE 3:**
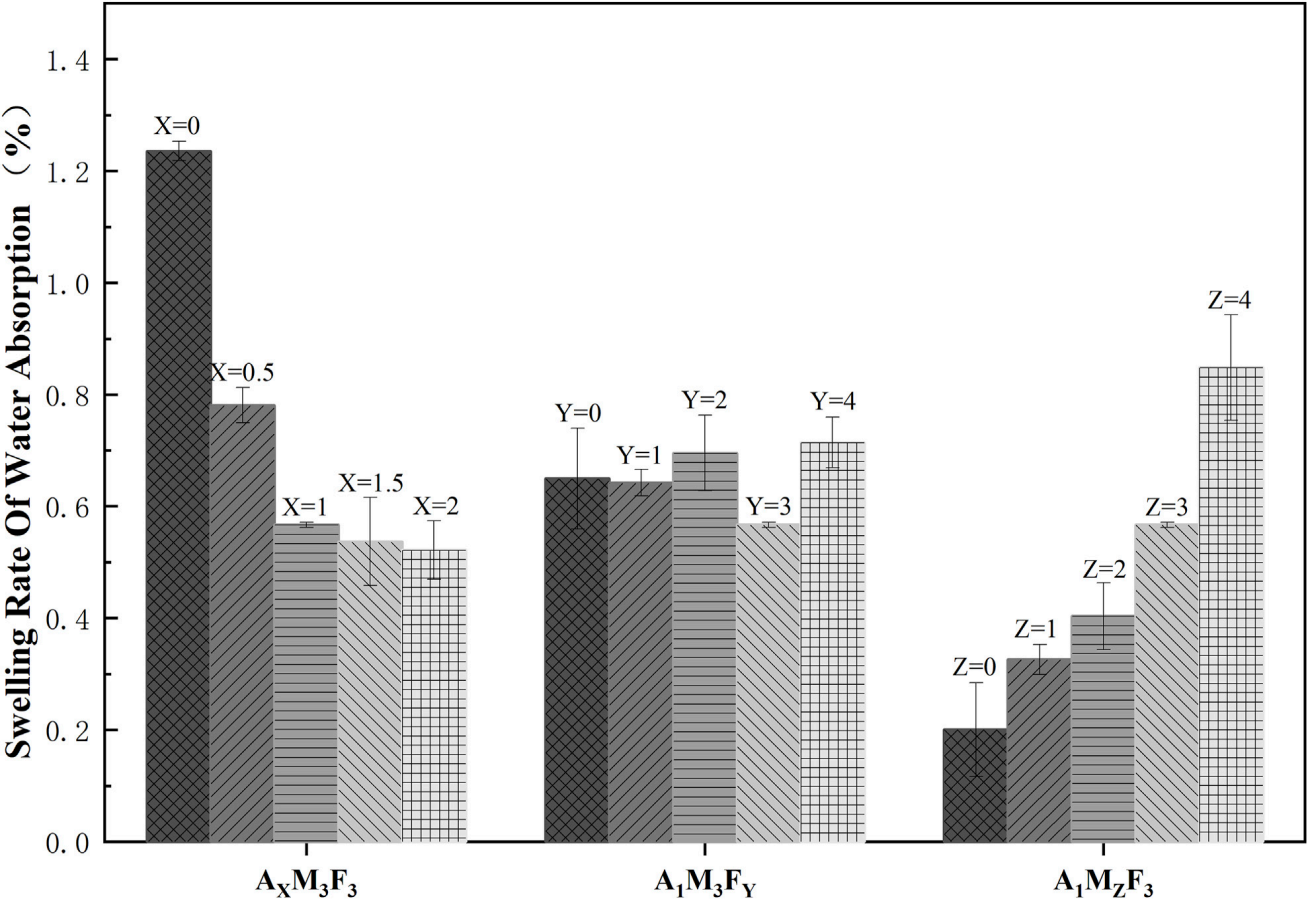
The rate of water absorption and swelling varies with the content of the substance.

To investigate the influence of A, M (MoS_2_), and F (PTFE) on coating hydrophilicity, we measured the water swelling ratio of various formulations (Figure 3). Three experimental series were examined: A_X_M_3_F_3_ Series (constant M/F at 3 wt%, varying A):When A content was 0, 0.5%, 1%, 1.5%, and 2%, the swelling ratios were 1.24%, 0.78%, 0.57%, 0.54%, and 0.52% respectively, demonstrating that hydrophobic A can effectively reduce the coating’s water absorption. A_1_M_3_F_Y_ Series (constant A/M at 1/3 wt%, varying F):With F content at 0%, 1%, 2%, 3%, and 4%, the swelling ratios were 0.65%, 0.64%, 0.70%, 0.57%, and 0.71% respectively, indicating F has negligible effect on water absorption within experimental error. A_1_M_Z_F_3_ Series (constant A/F at 1/3 wt%, varying M):When M content was 0%, 1%, 2%, 3%, and 4%, the swelling ratios were 0.20%, 0.33%, 0.40%, 0.57%, and 0.85% respectively, MoS_2_ increased from 0% to 4%, increasing the water absorption swelling rate by 325%. This may be attributed to microporosity formation in MoS_2_’s layered structure enhancing water absorption. Collectively, the introduction of A, F, and M components demonstrated limited influence on the coating’s water absorption properties, with the fundamental surface characteristics remaining largely unaffected.

### 3.3 Characterization of coating surface topography

**FIGURE 4:**
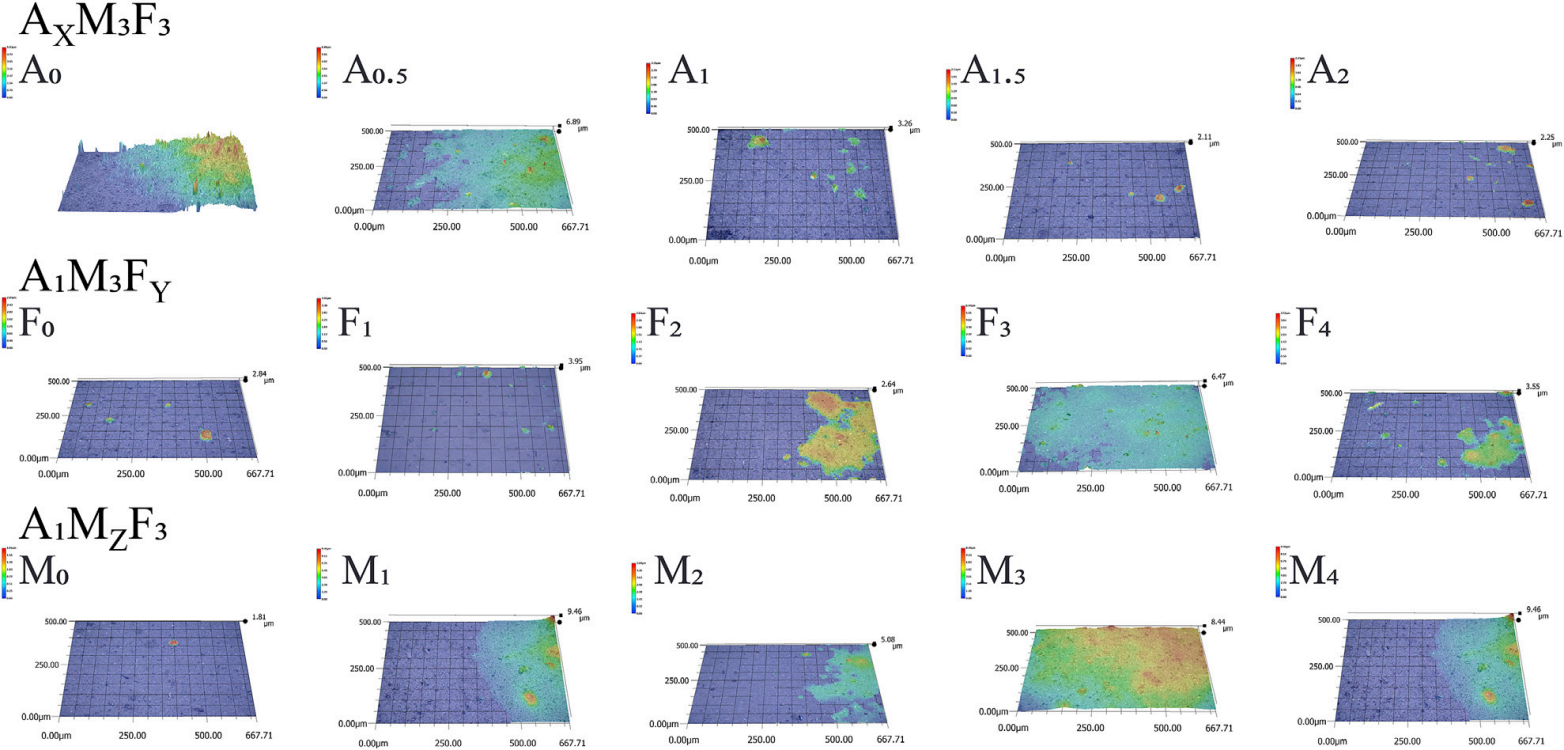
3D surface topography of coatings characterized by high-depth microscopy.

**FIGURE 5:**
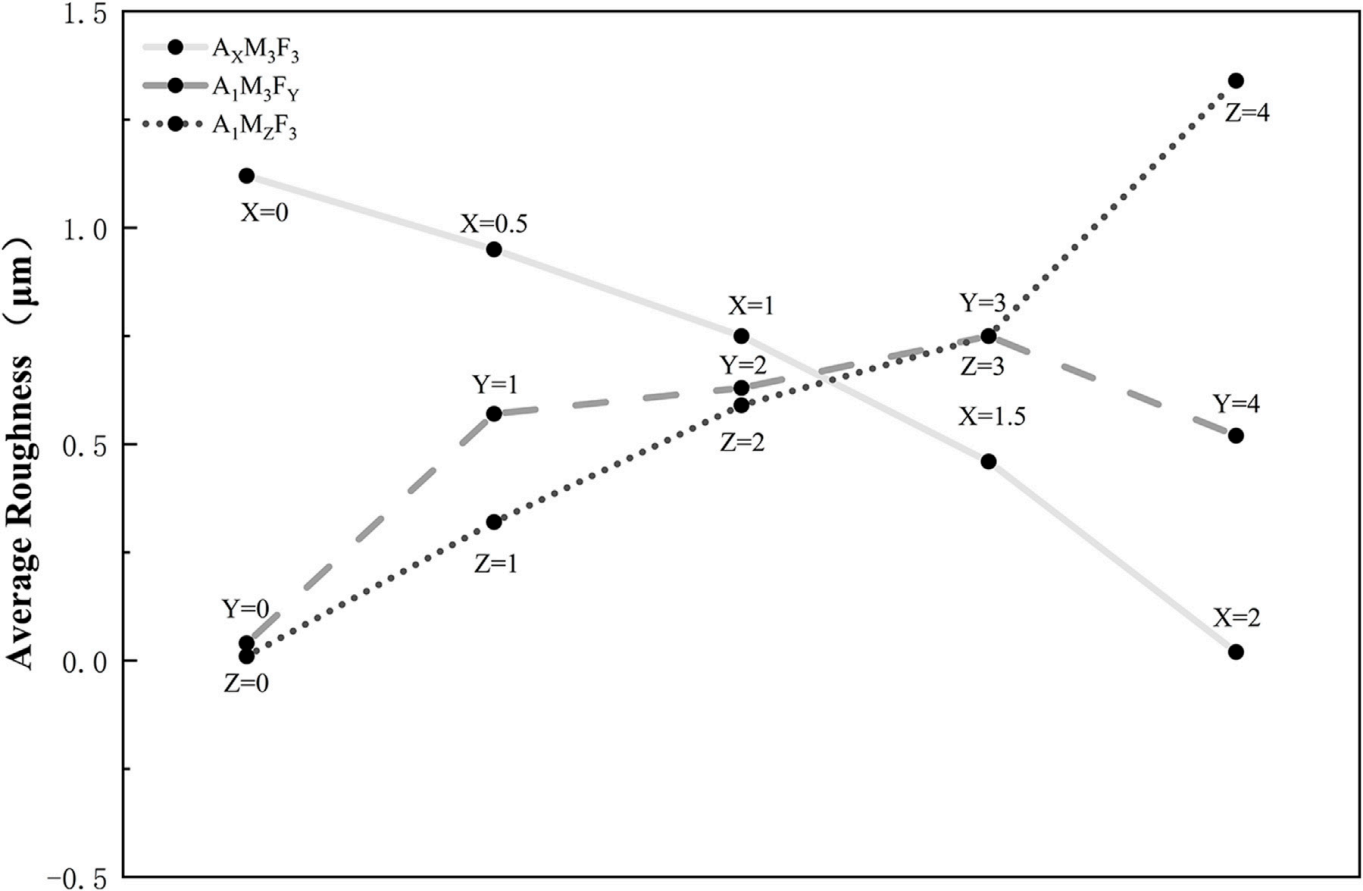
Dependence of surface roughness (Sa) on the composition of A, MoS_2_ (M), and PTFE (F) in silicone coatings.

As shown in Figures 4, 5, the surface morphology of gradient composition coatings was systematically characterized using ultra-depth three-dimensional microscopy to investigate the effects of component variations on surface topology. All coatings demonstrated excellent smoothness at both macro- and micro-scales, with average surface roughness (Sa) values below 5 μm across all samples. A_X_M_3_F_3_ Series (constant M/F at 3 wt%, varying A):With A content at 0, 0.5%, 1%, 1.5%, and 2%, the Sa values were 1.12, 0.95, 0.75, 0.46, and 0.02 μm respectively, showing a significant reduction in surface roughness with increasing amide content. A_1_M_3_F_Y_ Series (constant A/M at 1/3 wt%, varying F):When F content was 0%, 1%, 2%, 3%, and 4%, the Sa values measured 0.04, 0.57, 0.63, 0.75, and 0.52 μm respectively, exhibiting a general increasing trend with higher F loading. A_1_MzF_3_ Series (constant A/F at 1/3 wt%, varying M):With M content at 0%, 1%, 2%, 3%, and 4%, the Sa values were 0.01, 0.32, 0.59, 0.75, and 1.34 μm respectively, demonstrating a clear positive correlation between MoS_2_ content and surface roughness.

The results demonstrate that the incorporation of the amide component (A) significantly enhances the coating’s wear resistance, while increasing the content of MoS_2_ (M) and PTFE (F) moderately elevates surface roughness. This phenomenon may be attributed to slight phase separation between PTFE/MoS_2_ and the silicone matrix, leading to increased surface heterogeneity.

As shown in Figure 5, both A_1_M_3_F_4_ and A_1_M_4_F_3_ coatings demonstrated good wear resistance after 2000 abrasion cycles. For the A_1_M_3_F_4_ coating, the average surface roughness (Sa) measured after 500, 1,000, 1,500, and 2000 abrasion cycles was 0.52, 0.61, 0.8, 2.06, and 2.39 μm, respectively, with corresponding contact angles of 125.3°, 118°, 115°, 109°, and 103°. In comparison, the A_1_M_4_F_3_ coating exhibited Sa values of 0.02, 0.73, 1.05, 2.29, and 2.31 μm under the same cycling conditions. The water contact angles for this coating were 122.4°, 123°, 118°, 111°, and 109°, respectively.

High-depth microscopy analysis revealed that after 2,000 wear cycles, although the surface morphology underwent noticeable changes, the average roughness (Sa) remained below 2.65 μm—a relatively low value—further confirming that the amide modification effectively improves the coating’s wear resistance while maintaining surface integrity.

### 3.4 Surface wettability

**FIGURE 6:**
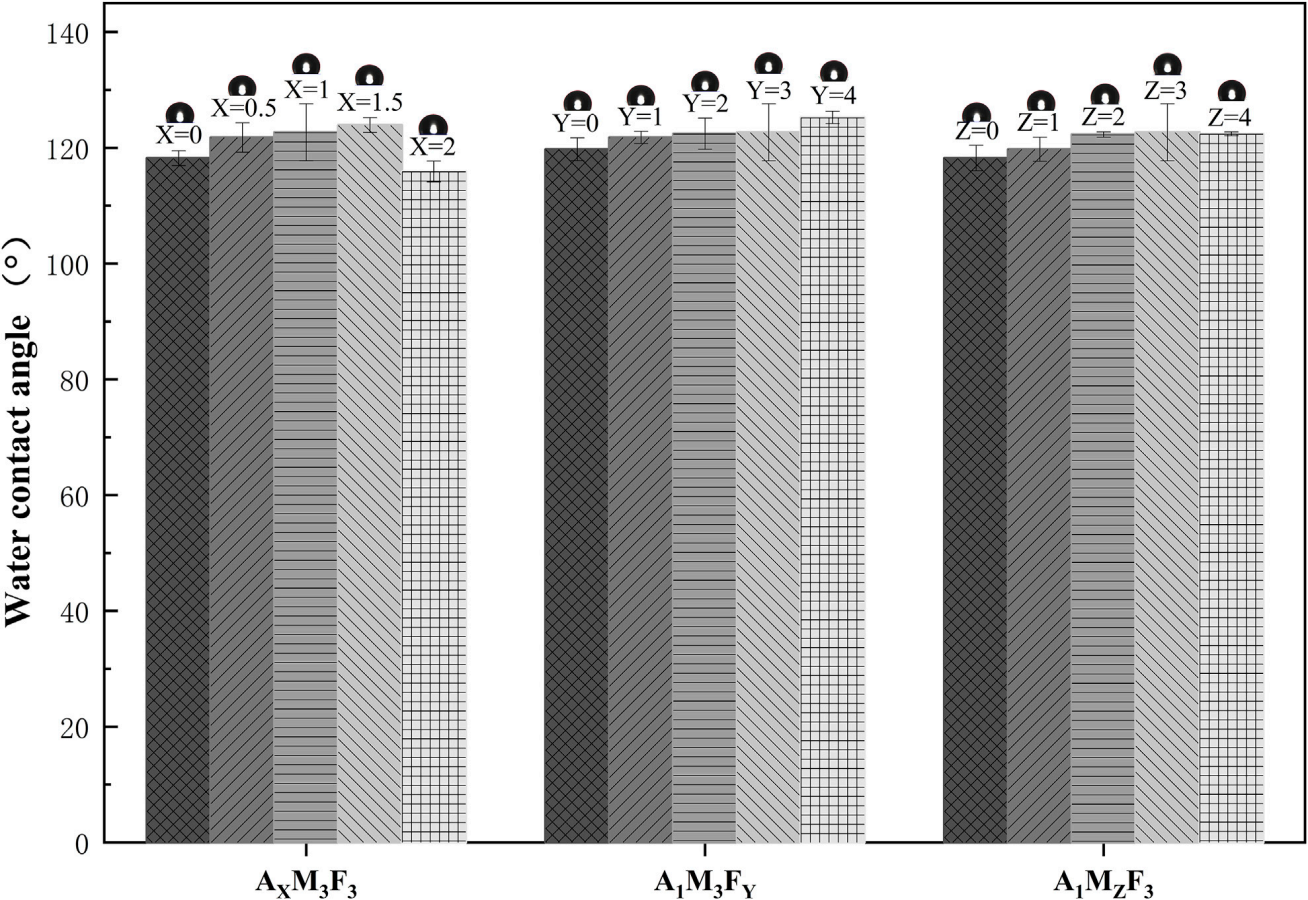
Water contact angle measurements of multicomponent coatings.

As illustrated in Figure 6, comprehensive water contact angle measurements were conducted to evaluate the surface wettability of the composite coatings under varying component ratios: In the A_X_M_3_F_3_ series (with both M and F contents fixed at 3%), when the A content was 0%, 0.5%, 1%, 1.5%, and 2% respectively, the surface contact angles were 118.2°, 121.8°, 122.7°, 123.9°, and 115.9° respectively. The contact angle showed an initial increase followed by a decrease. The maximum contact angle was achieved when the A content was 1.5%. The possible reason is that A acts both as a dispersant and a lubricant. At higher concentrations (2%), its surface migration characteristics become more pronounced, thereby reducing the surface contact angle of the coating. In the A_1_M_3_F_Y_ series (with A content at 1% and M content at 3%), when the F content was 0%, 1%, 2%, 3%, and 4% respectively, the surface contact angles were 119.8°, 121.8°, 122.4°, 122.7°, and 125.3° respectively. The contact angle gradually increased with increasing F content, which is consistent with other reported studies. In the A_1_M_Z_F_3_ series (with A content at 1% and F content at 3%), when the M content was 0%, 1%, 2%, 3%, and 4% respectively, the surface contact angles were 118.2°, 119.8°, 122.3°, 122.7°, and 122.4° respectively. The contact angle generally showed an increasing trend with increasing M content. It should be noted that when the M content exceeded 2%, the change in contact angle became almost negligible. From the data, it can be observed that all samples exhibited water contact angles (WCA) greater than 115°, demonstrating excellent hydrophobicity, which contributes to improved antifouling performance.

### 3.5 Underwater friction and wear testing

**FIGURE 7:**
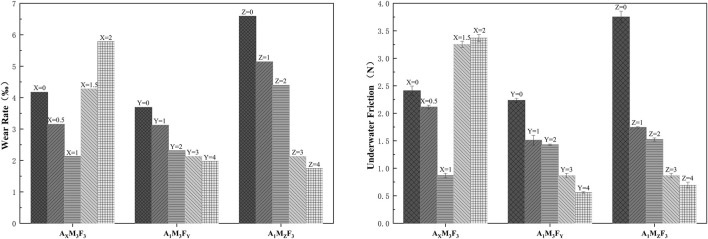
Quantitative Analysis of wear rate and underwater friction behavior in coatings.

As shown in Figure 7, the dynamic friction coefficients of the coatings under seawater conditions were measured using a tribometer equipped with an underwater testing module. The detailed results are as follows:

In the A_X_M_3_F_3_ series (with both M and F contents fixed at 3%), when the A content was 0%, 0.5%, 1%, 1.5%, and 2% respectively, the underwater friction forces were 2.41 N, 2.11 N, 0.87 N, 3.25 N, and 3.37 N respectively. The underwater friction force initially decreased and then increased with increasing A content. The possible reason is that an appropriate amount of amide can reduce surface roughness and act as a lubricant, while excessive amide may precipitate on the surface and consequently increase friction. In the A_1_M_3_F_Y_ series (with A content at 1% and M content at 3%), when the F content was 0%, 1%, 2%, 3%, and 4% respectively, the underwater friction forces were 2.24 N, 1.51 N, 1.43 N, 0.87 N, and 0.56 N respectively. The underwater friction force gradually decreased with increasing F content, demonstrating that F is effective in reducing friction. In the A_1_M_z_F_3_ series (with A content at 1% and F content at 3%), when the M content was 0%, 1%, 2%, 3%, and 4% respectively, the underwater friction forces were 3.75 N, 1.74 N, 1.52 N, 0.87 N, and 0.69 N respectively. The underwater friction force showed a decreasing trend with increasing M content, which may be attributed to the layered crystal structure of MoS_2_ that facilitates interlayer sliding and consequently reduces the friction coefficient when incorporated into the coating.

In our study system, the underwater friction force initially decreased and then increased with increasing A content, reaching its minimum value when the A content was 1%. Both M and F showed consistent trends, with the underwater friction force gradually decreasing as their contents increased.

According to Figure 7, the wear rate of the coating exhibits a positive yet nonlinear correlation with underwater friction. This nonlinearity arises from two competing mechanisms: (1) higher friction forces directly promote surface wear, whereas (2) the coating’s mechanical strength (reaching 2.23 MPa tensile strength at 1.5 wt% additive A) effectively resists wear progression.

When the PTFE content was 4 wt% (A_1_M_3_F_4_), compared with A_1_M_3_F_3_, the friction force drops to 0.56 N (a 35.4% reduction), but the wear rate decreased only marginally (7.0%) due to its lower mechanical strength (the elastic modulus declined by 12.5%). The interplay between mechanical properties and frictional behavior resulted in a piecewise linear trend in the wear rate-friction force relationship.

### 3.6 Mechanical properties testing

**FIGURE 8:**
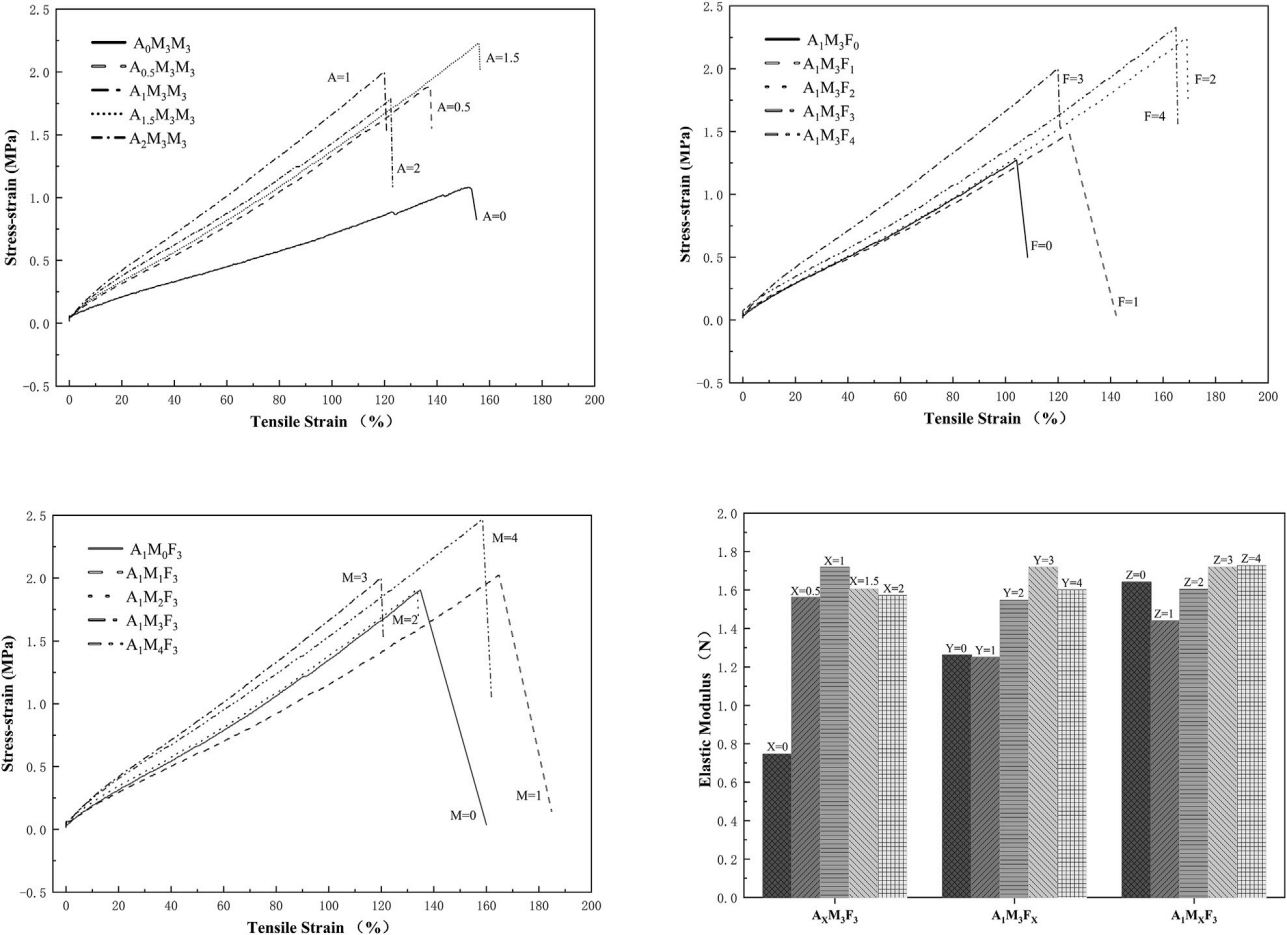
Mechanical performance optimization in A/M/F composite coatings.

As shown in Figure 8, the mechanical properties of all samples, including tensile strength and elongation at break, were evaluated through stress-strain curve measurements. The detailed results are presented as follows:

A_X_M_3_F_3_ Series (M and F fixed at 3 wt%, varying A), when the A content was 0%, 0.5%, 1%, 1.5%, and 2%, the tensile strengths were 1.08 MPa, 1.89 MPa, 2.00 MPa, 2.23 MPa, and 1.79 MPa, respectively. Even with only 0.5% A, the tensile strength increased by 75%, and the elastic modulus improved by 109%. At 1.5% A, the strength reached its maximum value (2.23 MPa), representing a 106% increase in tensile strength and a 115% increase in elastic modulus. These results demonstrate that the addition of A effectively enhances the mechanical properties of the coating. A_1_M_3_F_Y_ Series (A fixed at 1 wt%, M fixed at 3 wt%, varying F), when the F content was 0%, 1%, 2%, 3%, and 4%, the tensile strengths were 1.27 MPa, 1.48 MPa, 2.25 MPa, 2.00 MPa, and 2.33 MPa, respectively. At 1% F, the tensile strength increased by only 17%, with almost no change in elastic modulus. However, when the F content exceeded 2%, the tensile strength showed significant improvement, ranging from 57% to 83%, while the elastic modulus increased by 23%–36%. A_1_MzF_3_ Series (A fixed at 1 wt%, F fixed at 3 wt%, varying M), when the M content was 0%, 1%, 2%, 3%, and 4%, the tensile strengths were 1.91 MPa, 2.02 MPa, 1.91 MPa, 2.00 MPa, and 2.47 MPa, respectively. As the M content increased, the changes in tensile strength and elastic modulus were not significant, indicating that M does not substantially alter the mechanical properties of the material.

The synergistic mechanism among A, MoS_2_, and PTFE was crucial for achieving the coating’s exceptional wear resistance. Perhaps the amide group in A formed intermolecular hydrogen bonds with the resin matrix, MoS_2_, and cross-linking agents, effectively improving stress transfer efficiency and enhancing mechanical properties—resulting in an 85% increase in tensile strength. Meanwhile, the layered structure of MoS_2_ provided interlayer lubrication, significantly reducing frictional resistance, while PTFE’s low surface energy inhibited fouling adhesion, and its self-lubricating effect further minimized frictional losses. Their combined action reduced the underwater friction coefficient by 64% compared to the control sample. Additionally, the long alkyl chains in A improved the dispersion of MoS_2_ and PTFE through non-polar interactions, preventing agglomeration and enhancing distribution uniformity. This reduced stress concentration and resulted in a more homogeneous coating surface, with a 33% reduction in surface roughness. This ternary synergistic strategy successfully balanced mechanical durability (remaining intact after 2,000 friction cycles) and low-surface-energy characteristics (contact angles all exceeding 118°), providing a novel solution to the long-standing challenge of reconciling mechanical performance with low surface energy in traditional antifouling coatings.

### 3.7 Self-cleaning performance test

**FIGURE 9:**
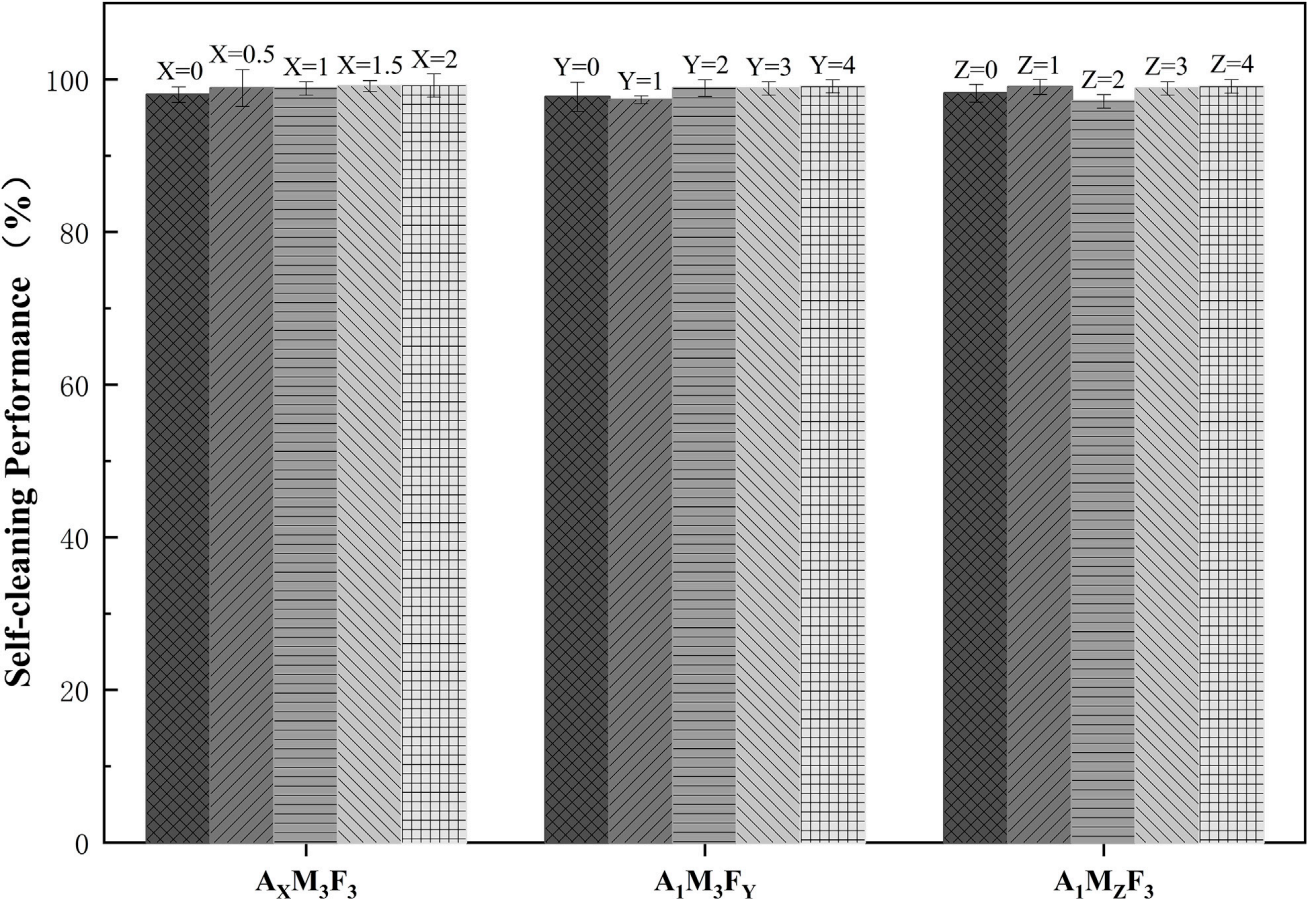
Self-cleaning performance of A/M/F composite coatings.

As shown in Figure 9, the self-cleaning efficiency of the coatings was systematically evaluated, with the following detailed results:

In the A_X_M_3_F_3_ series (with both M and F contents fixed at 3 wt%), when the A content was 0%, 0.5%, 1%, 1.5%, and 2% respectively, the self-cleaning efficiencies were 97.99%, 98.86%, 98.82%, 99.12%, and 99.21% respectively. The self-cleaning efficiency showed a slight increase with increasing A content. This may be attributed to the amide component reducing surface roughness and consequently decreasing pollutant adsorption, leading to a mild improvement in self-cleaning performance. In the A_1_M_3_F_Y_ series (with A content fixed at 1 wt% and M content at 3 wt%), when the F content was 0%, 1%, 2%, 3%, and 4% respectively, the self-cleaning efficiencies were 97.72%, 97.32%, 98.87%, 98.82%, and 99.08% respectively. The self-cleaning performance generally improved with increasing F content. In the A_1_M_Z_F_3_ series (with A content fixed at 1 wt% and F content at 3 wt%), when the M content was 0%, 1%, 2%, 3%, and 4% respectively, the self-cleaning efficiencies were 98.18%, 99.02%, 97.12%, 98.82%, and 99.08% respectively. The self-cleaning efficiency exhibited irregular variations with increasing M content, suggesting that the influence of M on self-cleaning performance may be complex. From the experimental data, it can be concluded that all samples demonstrated excellent self-cleaning efficiency (>97.1 ± 0.87%), indicating good antifouling potential of the coatings.

### 3.8 Testing of antimicrobial properties of coatings

**FIGURE 10:**
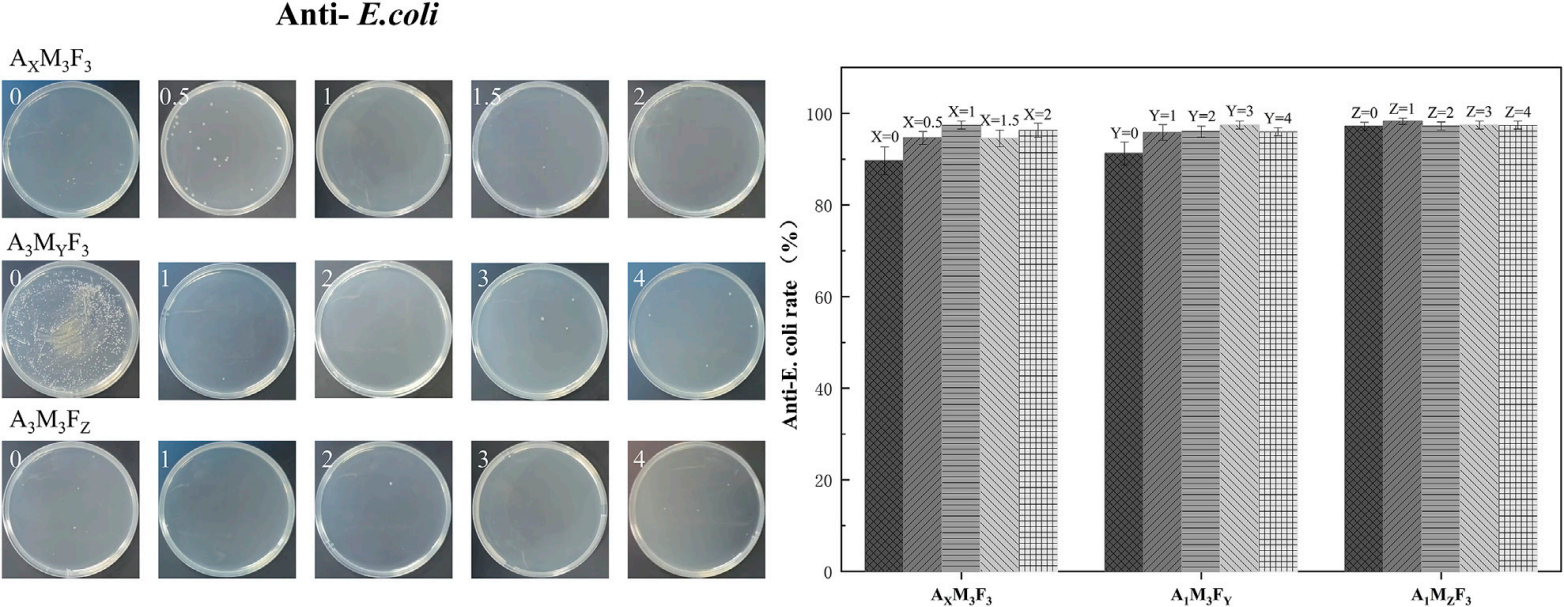
*E. coli* bacterial inhibition rate and bacterial inhibition experiments in real life graphs.

**FIGURE 11:**
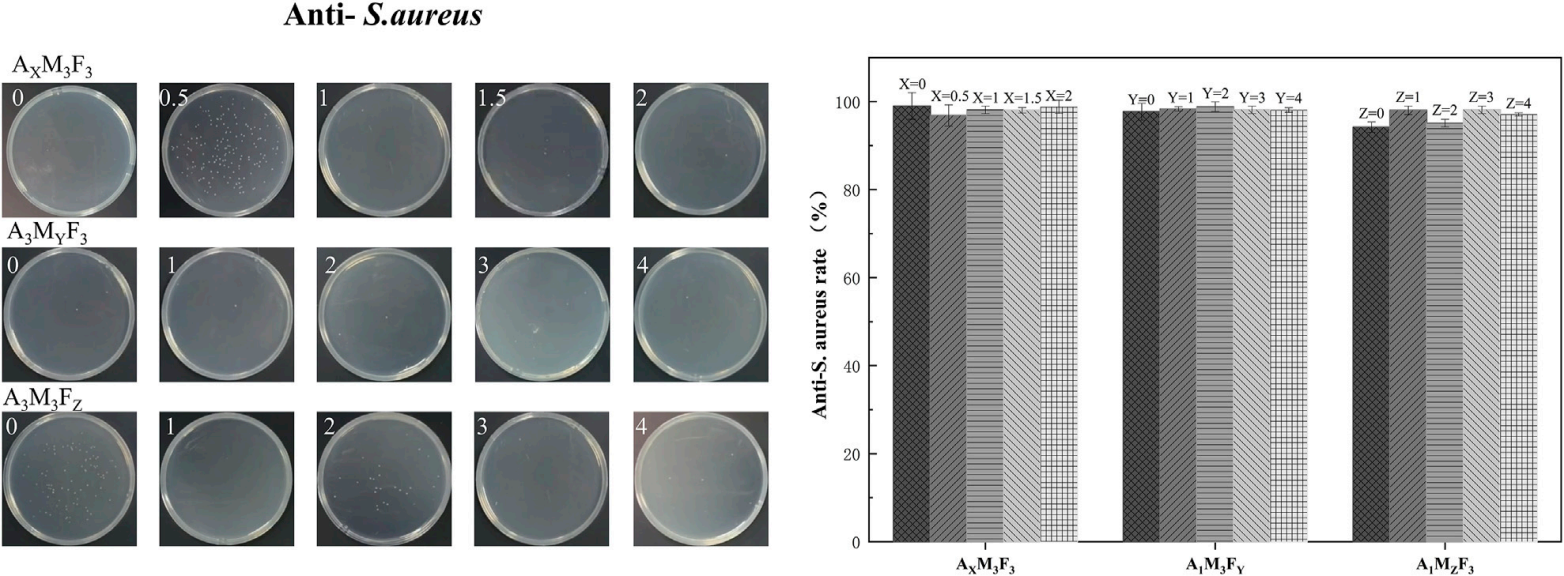
*S. aureus* bacterial inhibition rate and bacterial inhibition experiments in real life graphs.

As shown in Figures 10, 11, the antibacterial properties of the coatings were systematically evaluated against Gram-negative *E. coli* (*E. coli*) and *Staphylococcus aureus* (*S. aureus*) using the plate counting method. The detailed results are presented below.

#### 3.8.1 Anti-*E. coli*

In the A_X_M_3_F_3_ series (with both M and F contents fixed at 3 wt%), when the A content was 0%, 0.5%, 1%, 1.5%, and 2% respectively, the antibacterial rates against *E. coli* were 89.63%, 94.62%, 97.44%, 94.51%, and 96.33% respectively. The antibacterial rate showed wave-like variations but demonstrated an overall increasing trend with increasing A content. Notably, the addition of just 0.5% A improved the antibacterial rate by more than 5 percentage points. This enhancement may be attributed to A’s ability to reduce surface roughness, thereby inhibiting bacterial adhesion. In the A_1_M_3_F_Y_ series (with A content fixed at 1 wt% and M content at 3 wt%), when the F content was 0%, 1%, 2%, 3%, and 4% respectively, the antibacterial rates against *E. coli* were 91.24%, 95.83%, 96.04%, 96.36%, and 96.00% respectively. The antibacterial rate initially increased and then stabilized with increasing F content, showing no significant improvement when F content exceeded 1%. This suggests that low F content is sufficient to achieve optimal antibacterial effects. In the A_1_M_Z_F_3_ series (with A content fixed at 1 wt% and F content at 3 wt%), when the M content was 0%, 1%, 2%, 3%, and 4% respectively, the antibacterial rates against *E. coli* were 97.15%, 98.26%, 97.25%, 97.44%, and 97.44% respectively. The antibacterial rate remained essentially unchanged with increasing MoS_2_ content, indicating that MoS_2_ does not significantly affect the coating’s antibacterial properties.

#### 3.8.2 Anti-*S. aureusv*

In the A_X_M_3_F_3_ series (with both M and F contents fixed at 3 wt%), when the A content was 0%, 0.5%, 1%, 1.5%, and 2% respectively, the antibacterial rates against *S. aureus* were 98.99%, 96.86%, 98.12%, 98.02%, and 98.81% respectively. The antibacterial rate showed a slight decreasing trend with increasing A content. This contrasting trend compared to *E. coli* may be attributed to differences in metabolic pathways and cell wall structures between these two bacterial species. Overall, A’s effect on the coating’s anti-*S. aureus* performance appears relatively limited. In the A_1_M_3_F_Y_ series (with A content fixed at 1 wt% and M content at 3 wt%), when the F content was 0%, 1%, 2%, 3%, and 4% respectively, the antibacterial rates against *S. aureus* were 97.72%, 98.32%, 98.86%, 98.11%, and 98.08% respectively. The antibacterial rate remained consistently high without significant variation as F content increased. In the A_1_M_Z_F_3_ series (with A content fixed at 1 wt% and F content at 3 wt%), when the M content was 0%, 1%, 2%, 3%, and 4% respectively, the antibacterial rates against *S. aureus* were 94.18%, 98.02%, 95.12%, 98.12%, and 97.08% respectively. While the antibacterial rate showed irregular variations with increasing MoS_2_ content, all values demonstrated improvement compared to the baseline (0% M), suggesting that MoS_2_ incorporation does contribute to enhanced antibacterial performance.

### 3.9 Antifouling tests on shallow planks

**FIGURE 12:**
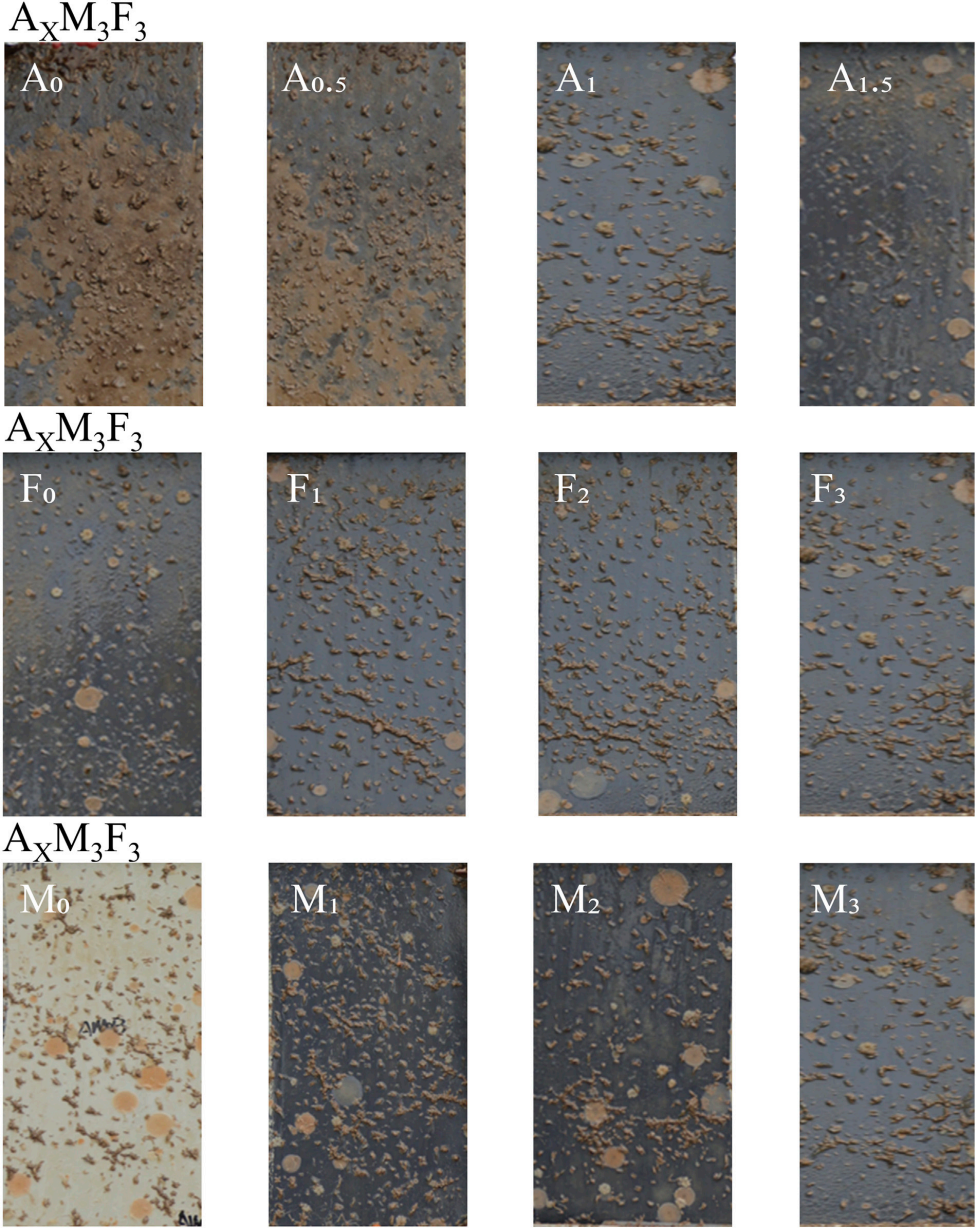
Marine field test of coatings in Zhoushan Sea.

As shown in Figure 12, real-world antifouling performance was evaluated through 3-month shallow sea panel tests conducted during peak marine organism growth season. The detailed results are presented below:

In the A_X_M_3_F_3_ series (with both M and F contents fixed at 3 wt%), when the A content was 0%, 0.5%, 1%, 1.5%, and 2% respectively, the fouling organism attachment on the coatings significantly decreased with increasing A content. This improvement may be attributed to A’s dual functionality as both dispersant and lubricant - as its concentration increased, both dispersion capability and surface smoothness were enhanced, thereby improving antifouling performance. In the A_1_M_Z_F_3_ series (with A fixed at 1 wt% and F at 3 wt%) and A_1_M_3_F_Y_ series (with A fixed at 1 wt% and M at 3 wt%), variations in M and F content showed no improvement in antifouling performance, with some samples even demonstrating reduced performance. This indicates that M and F do not significantly influence antifouling properties.

## 4 Summary

Through condensation reaction, we successfully synthesized a multifunctional anchoring material A incorporating long alkyl chains, polar amide groups, and benzene rings. This material was then blended with MoS_2_ and PTFE into a silicone resin system, followed by room-temperature crosslinking curing to prepare a novel antifouling coating. Under the synergistic effects of A, MoS_2_ and PTFE: Surface roughness was reduced below 2 μm, tensile strength exceeded 1.6 MPa for most coatings, reaching up to 2.47 Mpa. Average roughness remained below 2.65 μm after 2000 abrasion cycles, self-cleaning efficiency exceeded 97% and antibacterial rate surpassed 95%. The coatings maintained effective antifouling performance for over 90 days during peak marine organism growth season. Component A played a dominant role in mechanical property enhancement, while M and F were more effective in reducing underwater friction. The synergistic interaction among A, MoS_2_ and PTFE resulted in coatings with exceptional mechanical properties that not only meet the requirements for underwater cleaning applications but also provide new solutions for designing high-performance antifouling coatings.

## Data Availability

The original contributions presented in the study are included in the article/supplementary material, further inquiries can be directed to the corresponding author.
